# Spirobifluorene Core-Based Novel Hole Transporting Materials for Red Phosphorescence OLEDs

**DOI:** 10.3390/molecules22030464

**Published:** 2017-03-14

**Authors:** Ramanaskanda Braveenth, Hyeong Woo Bae, Quynh Pham Bao Nguyen, Haye Min Ko, Choong Hun Lee, Hyeong Jun Kim, Jang Hyuk Kwon, Kyu Yun Chai

**Affiliations:** 1 Division of Bio-Nanochemistry, College of Natural Sciences, Wonkwang University, Chonbuk, Iksan 570-749, Korea; braveenth.czbt@gmail.com (R.B.); quynhnpb2003@yahoo.com (Q.P.B.N.); hayeminko@wku.ac.kr (H.M.K.); 2 Department of Information Display, Kyung Hee University, Dongdaemoon-gu, Seoul 130-701, Korea; hwbae@khu.ac.kr; 3 Division of Microelectronics and Display Technology, Wonkwang University, Iksan 570-749, Korea; chlee@wku.ac.kr (C.H.L.); sarayeboa@naver.com (H.J.K.); 4 Solar Cell Research Institute, Next Generation Industrial Radiation Technology RIC, Wonkwang University, Iksan 570-749, Korea; 5 Department of Carbon Fusion Engineering, Wonkwang University, Iksan 570-749, Korea

**Keywords:** organic light emitting diodes, hole transporting materials, red phosphorescence, spirobifluorene

## Abstract

Two new hole transporting materials, named **HTM 1A** and **HTM 1B**, were designed and synthesized in significant yields using the well-known Buchwald Hartwig and Suzuki cross- coupling reactions. Both materials showed higher decomposition temperatures (over 450 °C) at 5% weight reduction and **HTM 1B** exhibited a higher glass transition temperature of 180 °C. Red phosphorescence-based OLED devices were fabricated to analyze the device performances compared to Spiro-NPB and NPB as reference hole transporting materials. Devices consist of hole transporting material as **HTM 1B** showed better maximum current and power efficiencies of 16.16 cd/A and 11.17 lm/W, at the same time it revealed an improved external quantum efficiency of 13.64%. This efficiency is considerably higher than that of Spiro-NPB and NPB-based reference devices.

## 1. Introduction

Organic light emitting diodes (OLEDs) have received much attention towards practical application from both the academic and industrial communities. This next generation display technology provides many benefits such as reduced power consumption, high contrast, wide view angles, high brightness and flexible displays [1,2,3]. General structure of OLEDs contains different layers like the hole transporting layer (HTL), emission layer (EML) and electron transport layer (ETL) between a transparent anode and cathode. Past studies have revealed that multilayer OLED device structures typically boost the performance of the devices by reducing the hurdle for hole and electron injection from the anode and cathode, respectively [4,5,6]. Hole transporting materials (HTMs) mainly play an important role in the characteristics of phosphorescent-based OLED devices because of their hole density, hole accumulation and electron blocking ability at the interfacial region between the emission layer and the hole transporting layer. To overcome these issues, hole transporting materials (HTMs) should possess good hole mobility, suitable frontier molecular orbitals (FMOs) for hole injection and electron blocking nature and possess high triplet energy for blocking triplet excitons. Moreover, hole transporting materials should exhibit higher glass transition temperatures in order to suppress the crystallization feature during device operation and also good film formation during fabrication processes [7,8,9,10].

Recently, significant improvements have been accomplished for thermally, morphologically and high hole mobility hole transporting materials by using electron donating moieties such as arylamines, carbazole-based derivatives and spiro-linked amines [11,12,13,14,15,16,17,18,19]. During the last few decades, the most widely used HTMs were 4,4′-bis(*N*-phenyl-1-naphthylamino)biphenyl (NPB), 4,4′,4″-tris(*N*-carbazolyl)-triphenylamine (TCTA), 4,4′-bis(3-methylphenylphenylamino)biphenyl (TPD) and di-[4-(*N*,*N*-ditolyl-amino)phenyl]cyclohexane (TAPC) for their considerable efficiencies, but they lack proper morphological stabilities, as indicated by low glass transition temperatures [4,20,21,22].

Initially spirobifluorene-based molecules were introduced as host materials and blue fluorescent emitters in several organic electronics devices to ensure the effective energy flow from the host to the guest system. The spirobifluorene core can provide certain characteristics like higher triplet energy, elevated glass transition temperature, stable decomposition temperature and matching of frontier molecular orbital (FMO) energy values with adjacent layers. The higher triplet energy of spirobifluorene caused by conjugation disconnection through its sp^3^ carbon and twisted non-coplanar structure can prevent the intermolecular interaction to support the stable film formation during the device fabrication process [23,24,25]. At the same time, the spirobifluorene core can offer the possibility of modification at the 2nd and 7th positions to obtain higher chemical reactivity [7,26]. The most commonly known spirobifluorene based HTM is *N*^2^,*N*^7^-di(naphthalen-1-yl)-*N*^2^,*N*^7^-diphenyl-9,9′-spirobi[fluorene]-2,7-diamine (Spiro-NPB) [27,28]. In this work, we have modified the spirobifluorene structure to produce an effective hole transporting material by attaching electron-rich nitrogen-based amine derivatives A and B, which increases the conjugation length of the molecules, which can reduce the energy level and ensure higher quantum efficiencies. The synthesized derivatives, named **HTM 1A** and **HTM 1B**, were applied on a red phosphorescence-based OLED device and we compared the device properties with the references Spiro-NPB and NPB. Here we adopt Spiro-NPB for our reference due to the similarity of its core structure with our designed materials. **HTM 1B** showed better device performances than reference materials and improved external quantum efficiencies up to 13.64%.

## 2. Results and Discussion

### 2.1. Synthesis

**Scheme 1:**
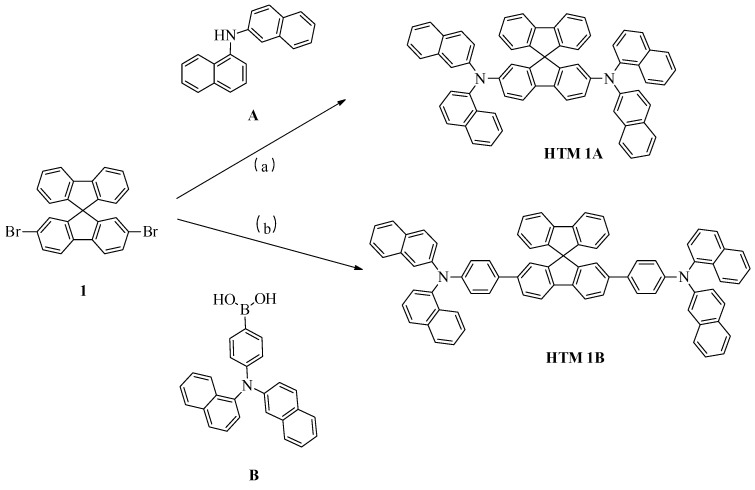
Synthesis of **HTMs 1A** and **1B**. *Reagents and Conditions*: (**a**) Pd(OAc)_2_, NaOtBu, *t*-Bu_3_P, anhydrous toluene, 105 °C; (**b**) Pd(Ph_3_P)_4_, 2 M K_2_CO_3_, toluene, 110 °C.

Scheme 1 displays the synthetic process of our designed spiro-based hole transporting materials (HTMs). Those materials were synthesized by using the well-known Buchwald Hartwig and Suzuki cross-coupling reaction between the derivatives 2,7-dibromo-9,9′-spirobi[fluorene] (**1**), *N*-(naphthalene-2-yl)naphthalene-1-amine (**A**) and 4-(*N*-(naphthalen-2-yl)-*N*-(naphthalen-4-yl)amino)phenylboronic acid (**B**) using a palladium-based catalyst. The designed HTMs were obtained with good yields of 74% and 64.4%, respectively, and revealed good solubility in most of the common organic solvents like dichloromethane and chloroform. The structures of the obtained compounds were confirmed by NMR spectroscopy (^1^H, ^13^C) and mass spectrometry.

### 2.2.Thermal Properties

Thermal properties were investigated using differential scanning calorimetry (DSC) and thermogravimetric analysis (TGA) experiments, carried out under a nitrogen atmosphere. Both synthesized **HTMs 1A** and **1B** exhibited high decomposition temperatures over 450 °C at 5% weight reduction (Table 1). **HTM 1B** in particular showed a higher glass transition temperature of 180 °C due to its rigid core structure and higher molecular weight. The values mentioned above can confer good thermal and morphological stabilities during device construction and operation processes.

### 2.3. Photophysical Properties

**Figure 1:**
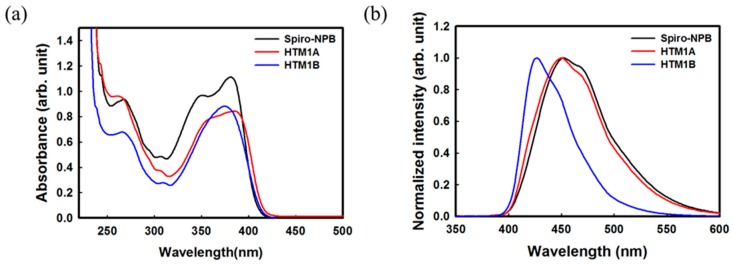
UV-visible absorption (**a**) and PL spectra (**b**) of **HTMs 1A**, **1B** and Spiro-NPB.

The photophysical properties were analyzed on the basis of the UV-visible spectra and photoluminescence spectra (PL) recorded at room and low (77 K) temperatures. Tetrahydrofuran (THF) was used as solvent for both measurements, which are depicted in Figure 1 and summarized in Table 1. 

**HTM 1A** and **HTM 1B** showed absorption wavelengths below 385 nm, meaning they have no absorption in the visible region. Absorption wavelength values are attributed to π-π* transitions of the conjugated aromatic rings. All materials showed little differences in their absorption patterns, but Spiro-NPB and **HTM 1A** revealed similar peak shapes. These similarities can be attributed to the similar type of amine directly attached with the central core, where in case of **HTM 1B** the central core and amine group are separated by a phenyl ring. We have observed there was a smaller absorption peak in all three molecules at 308 nm, which has been credited to the π-π* transition of the fluorene units in the spiro core [23,24]. Moreover, band gap values were obtained from the absorption spectra. All three spectra gave identical longer wavelength tail and similar bandgap energies so little difference was observed. The photoluminescence spectra of spiro-NPB and **HTM 1A** displayed similar emissions (450 nm) while **HTM 1B** showed a slightly blue-shifted emission. Triplet energy values were obtained from PL spectra, and were 2.31, 2.29 and 2.33 eV for **HTM 1A**, **HTM 1B** and Spiro-NPB. The triplet energy values were almost identical to each other, which is evidence for a similar localization of the triplet exciton. The spirobifluorene core has a higher triplet energy but here we obtained lower triplet energies through nitrogen enriched derivatives attached at the 2nd and 7th positions to ensure the effective hole transportation and extend the conjugation length of the molecules. When we compare the differences between absorption and emission, **HTM 1A** and **HTM 1B** showed smaller Stokes shift values of 65 nm and 52 nm, respectively.

### 2.4. Electrochemical Properties

**Figure 2:**
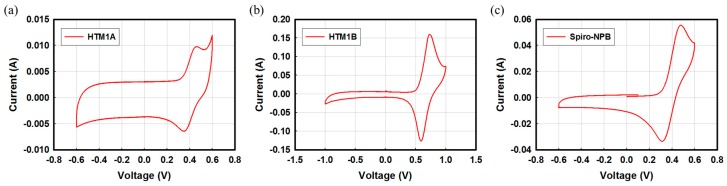
Cyclic voltammetry measurements of **HTMs 1A** (**a**), **1B** (**b**) and Spiro-NPB (**c**).

Highest occupied molecular orbital (HOMO) energy values were elucidated from cyclic voltammetry (CV) measurements and are shown in Figure 2 and summarized in Table 1. The calculated HOMO levels of **HTM 1A** and **1B** were −5.33 and −5.54 eV, with **HTM 1A** showing a slightly higher HOMO energy level than **HTM 1A** which can further facilitate its effective hole transport properties. 

**Figure 3:**
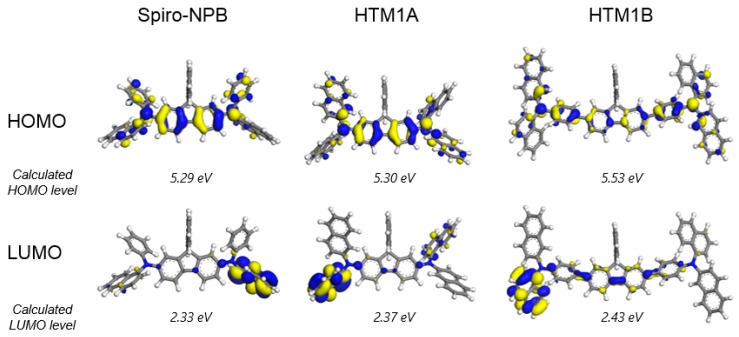
Calculated DFT frontier molecular orbitals of Spiro-NPB, **HTM 1A** and **HTM 1B**.

The above HOMO measurements were calculated by using the following equation: E_HOMO_ = −4.8 − (E_OX_ − E_Fc_) [29]. The incorporation of electron-rich donating moieties in the 2nd position of the fluorene ring can increase the HOMO values further. The spirobifluorene core has a HOMO value of 5.94 eV but our molecules exhibited higher HOMO energy levels due to the presence of the electron-donating derivatives **A** and **B** [23]. **HTM 1A** and Spiro-NPB showed similar HOMO energy levels around 5.32 eV since both of the derivatives are attached directly to the central core. In case of **HTM 1B**, the building blocks are separated by a phenyl ring from the central spiro core so there is a small change in the HOMO level (5.54 eV) due to the electron density difference when compared to the other materials. Lowest unoccupied molecular orbital (LUMO) energy values were calculated by subtraction of the energy gap values from the HOMO energy values. The resulting LUMO energy levels of both **HTM 1A** and **1B** were a little higher than that of the emission layer (EML) which is capable of electron blocking to prevent electrons from escaping from the emission layer. Additionally, we have done DFT calculations, depicted in Figure 3, to get a clear idea of the HOMO-LUMO distribution. The experimental frontier molecular orbital values were matched with DFT (density functional theory) calculations to substantiate our results. From the DFT simulation, we know that electrons were delocalized on the naphthalene group.

### 2.5. Device Performances

**Figure 4:**
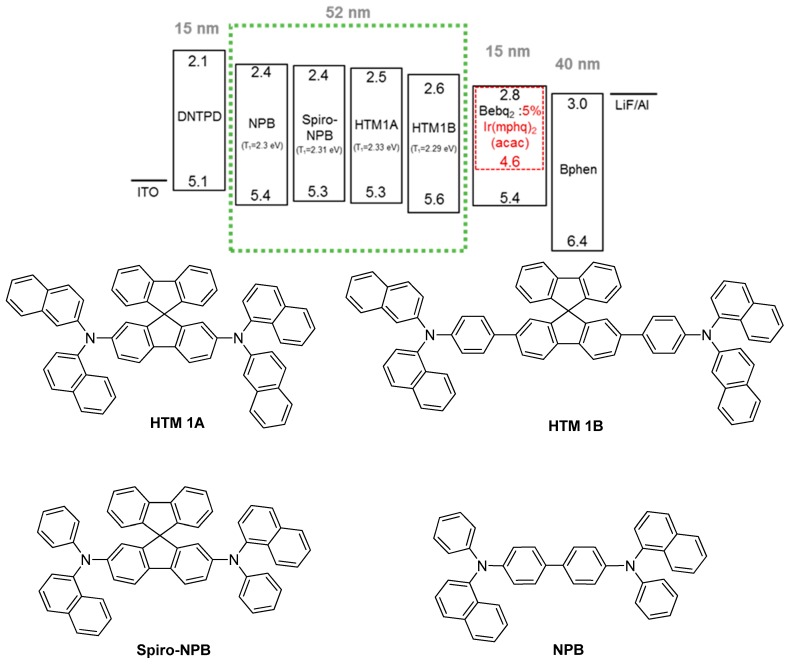
Configuration of red phosphorescent based OLED device and structure of hole transporting materials used for device fabrication.

In order to evaluate the performances of the synthesized HTMs, we have fabricated OLED devices based on red phosphorescence emitter. The fabricated device configuration is ITO (150 nm)/DNTPD (15 nm)/HTM (52 nm)/Bebq_2_: 5% Ir(mphq)_2_(acac) (15 nm)/Bphen (40 nm)/LiF (1 nm)/Al (100 nm), shown in Figure 4. 

**Figure 5:**
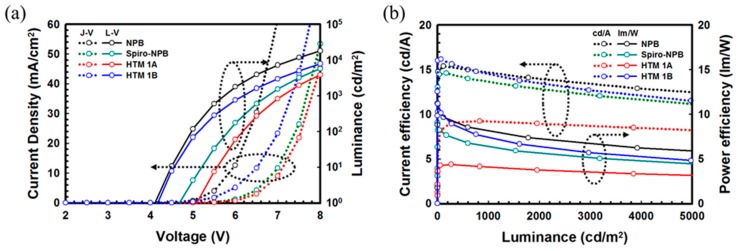
J-V-L (**a**) and luminescence-current efficiency characteristics (**b**) of the constructed OLED devices.

For further investigation of the performance of our designed materials, we have fabricated device 1 based on NPB and device II based on spiro-NPB as hole-transporting materials for our current studies. **HTM 1B**-based device IV and NPB-based device I showed similar turn on voltages of 4.0 V, which was lower than that of the spiro-NPB based device II (4.5 V) and which can boost the device efficiency. The current density-voltage-luminescence (J-V-L) characteristics and current efficiency spectra are shown in Figure 5 and summarized in Table 2. 

The maximum current efficiency of **HTM 1B**-based device IV was 16.16 cd/A and it was noticed that this value is higher than those of the references Spiro-NPB (9.24 cd/A) and NPB (15.38 cd/A). The device IV based on **HTM 1B** showed a more prominent power efficiency of 11.17 lm/W compared with Spiro-NPB (8.26 lm/W) because the **HTM 1B**-based device IV exhibited a lower driving voltage (6.2 V) than that of the reference-based device II (6.8 V). Consequently, the maximum external quantum efficiency of device IV was 13.64%, which was a superior value to that of Spiro-NPB-based device II (9.82%) and little higher than that of the NPB-based device I (13.58%).

**Figure 6:**
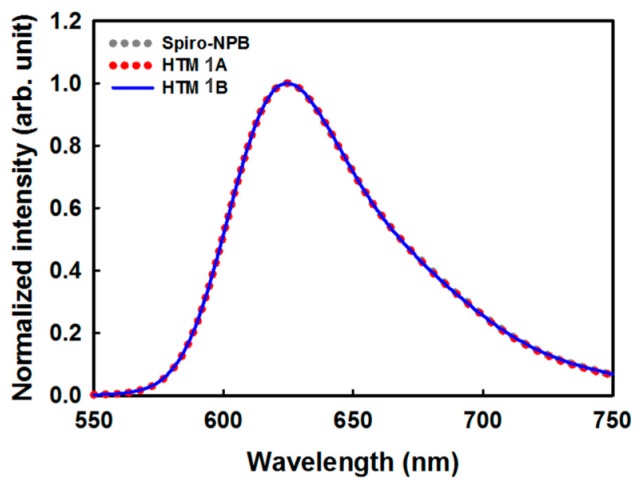
Normalized EL spectra of red phosphorescence based OLED devices.

Electroluminescent spectra (EL) of devices II to IV are displayed in Figure 6. All devices showed similar emissions and the results indicate that their emission was only limited to the emission layer and charge accumulation on the interfacial region is avoided.

## 3. Materials and Methods

### 3.1. General Procedures

All reagents and solvent were obtained from commercial suppliers and were used without further purification otherwise stated. ^1^H- and ^13^C-NMR spectra were recorded by using a JNM-ECP FT-NMR spectrometer (JEOL, Peabody, MA, USA) operating at 500 MHz. Absorbance spectra were obtained from a S-4100 UV-visible spectrophotometer (SINCO, Seoul, Korea). Band gaps (Eg) were estimated from the onset of the absorbance spectra. Photoluminescence (PL) spectra were measured using a FP-8500 spectrofluorimeter (JASCO, Tokyo, Japan) and tetrahydrofuran (THF) was used as a solvent. Triplet energy levels (ET) were determined by comparing the PL spectra recorded at room temperature and low temperature (~77 K). To measure the HOMO (highest occupied molecular orbital) and LUMO (lowest unoccupied molecular orbital) level, cyclic voltammetry (CV) was carried out by using a SP-50 system (BioLogic, Paris, France) and the HOMO level was calculated by subtracting the oxidation potential shift between ferrocene and the HTMs. LUMO level was estimated from the obtained HOMO level and band gap by adding them. Thermal gravimetric analysis was conducted on a DSC Q200 V24.9 Build 121 thermal analysis system (TA instruments, New castle, DE, USA) at a heating rate of 10 °C·min^−^^1^. Molecular simulations were carried by using density function theory (DFT) calculation with B3LYP. Mass analysis were carried out by using a Xevo TQ-S spectrometer (Waters, Milford, MA, USA).

### 3.2. Synthesis

#### 3.2.1. *N*^2^,*N*^7^-Di(naphthalen-1-yl)-*N*^2^,*N*^7^-di(naphthalen-2-yl)-9,9′-spirobi[fluorene]-2,7-diamine (**HTM 1A**)

A mixture of 2,7-dibromo-9,9′-spirobifluorene (**1**, 1 g, 1 equiv.) , *N*-(naphthalene-2-yl)naphthalene-1-amine (**A**, 1.6 g, 2.5 equiv.), Pd(OAc)_2_ (0.1 g, 0.2 equiv.), *t*-Bu_3_P (50% in toluene, 0.5 mL, 0.3 equiv.), NaO*t*-Bu in THF (2 M, 3.0 mL) and anhydrous toluene (100 mL) were mixed and then stirred under inert conditions at 105 °C for 24 h. After completion of the reaction, the reaction mixture was extracted with dichloromethane and water. The organic layer was dried over anhydrous magnesium sulphate then filtered and concentrated by rotary evaporation. The crude residue was purified by silica gel column chromatography by using a *n*-hexane−dichloromethane solvent system to provide the desired product **HTM 1A***.* Yield: 74%; yellow solid; ^1^H-NMR (CDCl_3_) δ 7.81–7.83 (d, *J* = 10 Hz, 2H), 7.75–7.77 (d, *J* = 10 Hz, 2H), 7.69–7.71 (d, *J* = 10 Hz, 2H), 7.59–7.61 (d, *J* = 10 Hz, 2H), 7.55–7.57 (d, *J* = 10 Hz, 2H), 7.44–7.49 (m, 4H), 7.37–7.39 (t, *J* = 9 Hz, 4H), 7.14–7.37 (m, 16H), 6.99–7.01 (t, *J* = 10 Hz, 2H), 6.89–6.91 (d, *J* = 10 Hz, 2H), 6.79–6.81 (d, *J* = 10 Hz, 2H), 6.63 (s, 2H); ^13^C-NMR (CDCl_3_) δ 108.1, 109.4, 121.4, 124.6, 125.0, 125.3, 127.5, 128.4, 133.7, 135.8, 140.1, 142.0, 142.4; GC-MS: 851.04 for C_65_H_42_N_2_ [M + H^+^].

#### 3.2.2. *N*,*N*′-(9,9′-Spirobi[fluorine]-2,7-diylbis(4,1-phenylene))bis(*N*-(naphthalene-2-yl)naphthalene-1-amine) (**HTM 1B**)

A mixture of 2,7-dibromo-9,9′-spirobifluorene (**1**, 1 g, 1 equiv.), 4-(*N*-(naphthalen-2-yl)*-N*-(naphthalen-4-yl)amino)phenylboronic acid (**B**, 2.5 g, 3 equiv.), Pd(Ph_3_P)_4_ (0.09 g, 0.2 equiv.), K_2_CO_3_ (2 M, 50 mL) and toluene (100 mL) were mixed and then stirred at 110 °C for 24 h. After completion of the reaction, the reaction mixture was extracted with dichloromethane and water. The organic layer was dried over anhydrous magnesium sulphate then filtered and concentrated by rotary evaporation. The crude residue was purified by silica gel column chromatography using a *n*-hexane−dichloromethane solvent system to provide pure **HTM 1B**. Yield: 64.4%; yellow solid; ^1^H-NMR (CDCl_3_) δ 7.81–7.90 (m, 8H), 7.75–7.77 (d, *J* = 10 Hz, 2H), 7.69–7.71 (d, *J* = 10 Hz, 2H), 7.63–7.65 (d, *J* = 10 Hz, 2H), 7.56–7.58 (d, *J* = 10 Hz, 2H), 7.40–7.48 (m, 6H), 7.24–7.35 (m, 18H), 7.07–7.10 (t, *J* = 11 Hz, 2H), 6.96–6.98 (d, *J* = 10 Hz, 4H), 6.88 (s, 2H), 6.78–6.80 (d, *J* = 10 Hz, 2H); ^13^C-NMR (CDCl_3_) δ 117.9, 120.0, 120.2, 122.0, 122.1, 122.9, 124.2, 124.3, 124.4, 126.2, 126.3, 126.4, 126.5, 126.6, 126.7, 126.9, 127.2, 127.5, 127.7, 127.9, 128.4, 128.8, 129.6, 131.1, 134.3, 134.4, 135.3, 140.2, 140.3, 141.8, 143.4, 145.9, 147.6, 149.8; GC-MS: 1003.05 for C_77_H_50_N_2_ [M + H^+^].

### 3.3. OLED Fabrication and Characterization

Red color OLED devices were fabricated for evaluation of the performance of the HTMs. An ITO substrate of 150 nm thickness was used as an anode electrode. Substrates were ultrasonicated with acetone, isopropyl alcohol, and deionized water and treated with ultraviolet-ozone. 4,4′-Bis[*N*-[4-{*N*,*N*-bis(3-methylphenyl)amino}phenyl]-*N*-phenylamino] biphenyl (DNTPD) was used as hole injection material. NPB and spiro NPB were used as a reference HTMs to compare with the synthesized HTMs. 5 wt % of iridium(III) bis(2-(3,5-dimethylphenyl)quinolinato-*N*,C2′)acetylacetonate (Ir(mphq)_2_(acac)) doped bis(10-hydroxybenzo[*h*]quinolinato)beryllium (Bebq_2_) was applied as EML host. Bathophenanthroline (bphen) was introduced as an electron injection layer. Aluminum with LiF was used as a cathode. Organic layers and cathode were thermally deposited in an evaporator system under ~1.0 × 10^−7^ torr pressure. Fabricated devices were encapsulated by glass caps. Current density-voltage-luminance (J-V-L) characteristics were estimated by a luminance and color meter (Konica Minolta CS-100A, Osaka, Japan) with a 2635A source meter unit (Keithley, Cleveland, OH, USA). A spectroradiometer (Konica Minolta CS-2000) was used to obtain electroluminescence (EL) spectra and Commission International l′Eclairage (CIE) 1931 color coordinators.

## 4. Conclusions

In this work, two new hole transporting materials named **HTMs 1A** and **1B** were designed, synthesized and characterized for application in red phosphorescence-based OLEDs. **HTM 1B** exhibited higher decomposition and transition glass temperatures (495 °C, 180 °C), which facilitated its good thermal and morphological stabilities. Device IV based on **HTM 1B** showed a higher current (16.16 cd/A) and power efficiency (11.17 lm/W) than our reference devices I and II. At the same time the **HTM 1B**-based device IV revealed a superior external quantum efficiency of 13.64%. Overall device performances were little higher than those of the NPB-based device I and superior to the spiro-NPB- based device II. We believe that **HTM 1B** could be a promising candidate for future applications in OLEDs.

## Figures and Tables

**Table 1:** Thermal and photophysical properties of **HTMs 1A**, **1B** and Spiro-NPB.

HTMs	*T_g_* ^a^ (°C)	*T_d_* ^b^ (°C)	UV-Vis (nm)	PL Max (nm)	HOMO (eV)	LUMO (eV)	E_g_^c^ (eV)	E_T_^d^ (eV)
**HTM 1A**	110	450	385	450	5.33 5.30 ^e^	2.45 2.37 ^e^	2.88 2.93 ^e^	2.31
**HTM 1B**	180	495	374	426	5.54 5.53 ^e^	2.62 2.43 ^e^	2.92 3.10 ^e^	2.29
Spiro-NPB	126	420	381	451	5.32 5.29 ^e^	2.38 2.33 ^e^	2.94 2.96 ^e^	2.33

^a^ Transition glass temperature, ^b^ Decomposition temperature at 5% weight reduction, ^c^ Band gap energy, ^d^ Triplet energy, ^e^ Calculated from DFT.

**Table 2:** Device characteristics.

Characteristics	Device I (NPB)	Device II (Spiro-NPB)	Device III (HTM 1A)	Device IV (HTM 1B)
Turn-on voltage (V)	4.0	4.5	5.0	4.0
Driving voltage ^a^ (V)	5.8	6.8	7.0	6.2
Current efficiency (cd/A)	14.68 ^a^	13.63 ^a^	9.19 ^a^	14.44 ^a^
	15.38 ^b^	14.60 ^b^	9.24 ^b^	16.16 ^b^
Power efficiency (lm/W)	8.16 ^a^	6.37 ^a^	4.38 ^a^	7.47 ^a^
	10.31 ^b^	8.26 ^b^	4.38 ^b^	11.17 ^b^
Max EQE	13.58%	9.82%	7.14%	13.64%
CIE 1931 (x, y) ^b^	(0.6688, 0.3302)	(0.6690, 0.3301)	(0.6641, 0.3349)	(0.6688, 0.3302)

^a^ Measured at 1000 cd/m^2^, ^b^ Maximum efficiency.
